# Vaccine Confidence and Vaccine Hesitancy in Several Countries in Southeastern Europe in Past 10 Years: A Structured Review of Published Literature

**DOI:** 10.3390/vaccines14040299

**Published:** 2026-03-27

**Authors:** Kaja Damnjanović, Kalin Djurov, Matea Galic, Bogdan Lisul, Ionut Viorel Mocanu, Shreya Shukla, Ashley Enstone, Lisa Dai, Mitja Vrdelja, Hristiana Batselova, Anca Drăgănescu, Goran Tešović

**Affiliations:** 1 Department of Psychology, University of Belgrade, 11000 Belgrade, Serbia; 2 MSD Bulgaria, 55 Nikola Vaptzarov Blvd.,1407 Sofia, Bulgaria; 3 MSD Croatia, 10010 Zagreb, Croatia; 4 MSD Serbia, 11070 Belgrade, Serbia; 5 MSD Romania, 013704 Bucharest, Romania; 6 Adelphi Values PROVE, Bollington SK10 5JB, UK; 7 National Institute of Public Health, 1000 Ljubljana, Slovenia; 8 Department of Epidemiology and Disaster Medicine, Medical University of Plovdiv, 4002 Plovdiv, Bulgaria; 9 National Institute for Infectious Diseases, 050463 Bucharest, Romania; 10 Pediatric Infectious Diseases Department, University Hospital for Infectious Diseases, University of Zagreb, School of Medicine, 10000 Zagreb, Croatia

**Keywords:** vaccine hesitancy, confidence, barriers to vaccination, impact of COVID-19, trust in vaccines, Southeastern Europe

## Abstract

Objectives: Despite vaccination being the most effective way of preventing infections and vaccination rates recovering worldwide after the COVID-19 pandemic, vaccine hesitancy persists. Some factors, such as psychological and social barriers, can negatively impact views on vaccines and can contribute to vaccine hesitancy. The primary objective of this structured literature review is to investigate the available evidence relating to factors affecting vaccine hesitancy within several countries in Southeastern Europe. Methods: An electronic database search was conducted to identify studies assessing the public and healthcare professionals’ (HCPs) attitudes towards vaccination in Southeastern Europe. These searches were supplemented with grey literature searches. Included studies were conducted in Bulgaria, Croatia, Romania, Serbia, and Slovenia between 1 January 2012 and 31 December 2022. Results: Of the 35 studies identified from the database searches, the most prominent theme observed across Romania, Croatia, and Bulgaria was low confidence in COVID-19 vaccines. Across all age groups, COVID-19 vaccine confidence in these regions was highly dependent on whether individuals thought vaccines were safe and effective, as well as their general trust in vaccines. Confidence in COVID-19 vaccines was seen as relatively high, with attitudes towards routine and elective vaccines being generally positive amongst the general public and HCPs, in Romania, Croatia, Serbia and Slovenia. However, uncertainty around the effectiveness of the vaccine still exists. In Bulgaria, trust in routine and elective vaccines remained low in the general public. Complacency and financial constraints were also identified as underlying causes of vaccine hesitancy. Conclusions: The main cause behind vaccine hesitancy in several countries in Southeastern Europe is distrust in vaccine effectiveness and safety. These key findings can be utilised to support evidence-based decisions regarding where to focus resources to improve public and HCP perception of vaccines in Southeastern Europe.

## 1. Introduction

Vaccinations are globally recognised as the most effective way of preventing infectious diseases and as a key strategy for improving health outcomes and life expectancy [1,2]. Evidence suggests that mass immunisation has greatly contributed to the 55.0% decrease in mortality of children aged less than 5 years since 1990 [3]. Additionally, vaccination campaigns aided in preventing 10 million deaths between 2010 and 2015 alone [4]. However, vaccine hesitancy—the decision to delay or refuse vaccination—is a significant obstacle to the success of ongoing immunisation schedules, with the World Health Organisation (WHO) declaring vaccine hesitancy as a ‘top 10 health threat’ [5,6,7,8].

While vaccine hesitancy is a global concern, low vaccine acceptance due to vaccine hesitancy has resulted in a resurgence of vaccine-preventable diseases such as measles across countries in Southeastern Europe [9,10]. In 2022, it was identified that Serbia’s national coverage with the first dose of the measles vaccine was 81%. However, the WHO recommends at least 95% coverage to prevent outbreaks of measles [11]. Furthermore, the European Centre for Disease Prevention and Control (ECDC) reported that in 2023, among the 2361 measles cases reported to the ECDC by countries in the European Union (EU) and European Economic Area (EEA), 1755 (74.3%) were reported by Romania, leading to the Romanian Ministry of Health declaring a national measles epidemic in December 2023 [12].

Uptake of the primary course of the COVID-19 vaccine in Southeastern Europe has also lagged behind the EU’s average of 73%. As of 2023, the uptake was at 30% in Bulgaria, 42% in Romania and 56% in Croatia and Slovenia [13]. Data has also shown that uptake of the COVID-19 vaccine was at 48% in Serbia. While mandatory vaccination in some EU countries may explain why the EU vaccine uptake is higher, vaccine hesitancy has been identified as a factor for low vaccine acceptance in Southeastern Europe [14].

With vaccine acceptance lagging and resurgence of vaccine-preventable diseases, particularly in countries such as Romania, Croatia, Bulgaria, Serbia and Slovenia, greater understanding of underlying causes of vaccine hesitancy within Southeastern Europe is required to devise methods to reduce vaccine hesitancy and increase vaccine confidence, which is the belief that vaccines work, are safe, and are part of a trustworthy medical system [15,16,17,18]. While reports have suggested that in Southeastern European countries, such as Bulgaria and Romania, there has been little trust in the government, which has led to vaccine rollout lagging behind most of Europe, vaccine hesitancy can vary by time, place, vaccine, subgroup, and person; therefore, understanding how each of these factors influences vaccine confidence is crucial [15,19].

Our structured literature review addressed the following research questions:

1. What are the key concerns and attitudes toward adult and paediatric vaccinations among healthcare professionals (HCPs) and the general public, including parents in several Southeastern European countries (Croatia, Serbia, Slovenia, Romania, and Bulgaria), and how do these attitudes differ by stakeholder group?
2. How do different sources of vaccine information influence attitudes towards vaccination?
3. How do attitudes differ between paediatric and adult immunisation?
4. How have beliefs in vaccination changed following the COVID-19 pandemic?

## 2. Materials and Methods

This study was conducted following the principles of a structured literature review and is presented in accordance with the Preferred Reporting Items for Systematic Reviews and Meta-Analyses (PRISMA) guidelines [20,21]. The Population, Intervention, Comparison, Outcomes, Time, and Study design (PICOTS) criteria were developed based on the objectives of the structured literature review (outlined in Supplementary Materials [Table S1]). The 5C model was also utilised as the theoretical framework to develop the PICOTS and categorise vaccine hesitancy-related data [22,23]. This model was developed by Betsch et al. and is based on established theoretical frameworks on health behaviour and factors of vaccination. This model measures 5 psychological antecedents of vaccination: confidence, complacency, constraints, calculation, and collective responsibility (outlined in the Supplementary Materials [Table S2]) [22].

### 2.1. Search Strategy

A comprehensive electronic search was conducted on 1 December 2022, going back to 1 January 2012, using Medline, Embase and Evidence-Based Medicine Reviews (EMBR). The search terms utilised are shown in the Supplementary Materials (Table S3). This period was chosen to capture the different perspectives of key stakeholders and how these may have changed over time, especially after the COVID-19 pandemic.

To gain comprehensive insight into the matter, the database searches were supplemented by grey literature searches provided by key opinion leaders (KOLs). In this structured literature review, grey literature sources included: government reports; PhD theses; European surveillance programme websites; country-specific public health websites; and social media channels reporting on vaccine hesitancy. Grey literature sources were available in English, Serbian, Romanian, Croatian, Slovenian and Bulgarian languages. Where possible, sources in local languages were translated using Google Translate.

### 2.2. Study Selection and Data Extraction

**Figure 1:**
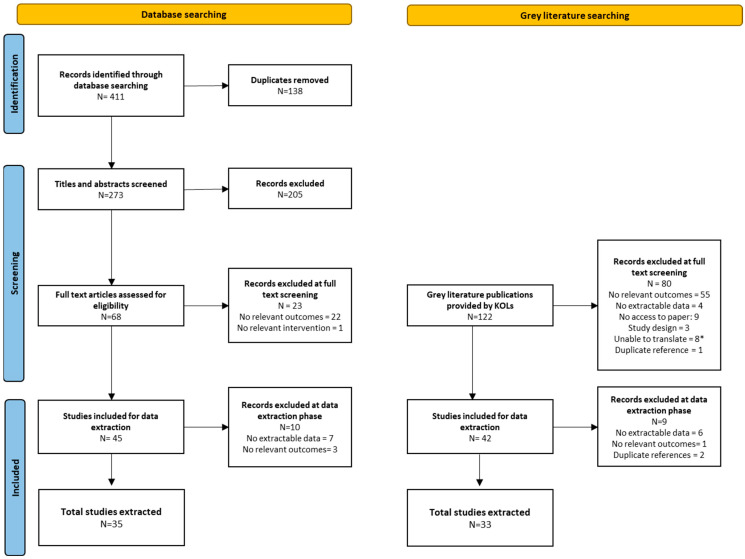
PRISMA flowchart of publications included in the structured literature review. * Sources considered non-translatable were social media platforms that included video.

Following the removal of duplicates, title and abstract screening was conducted on DistillerSR, which is literature review management software. Title and abstracts were screened against the inclusion and exclusion criteria, as outlined in Table S1. Included publications were screened based on the full text. Studies included as part of the full text screening were data-extracted. See Figure 1 for an overview of the publication inclusion process.

As per the principles of structured literature reviews, study selection was conducted by one researcher. In the screening process, the researcher was responsible for reviewing each publication. One senior researcher was responsible for reviewing the study selection decisions made to ensure quality and consistency.

Data was extracted into Microsoft Excel by one researcher and quality checked by a senior researcher. Study characteristics such as study design and data source were extracted, along with population characteristics, such as age range, education level, ethnicity, and vaccination history. Outcome data were categorised according to the 5C model. Within the data, various themes relating to the 5 psychological antecedents were identified. Further subcategories were developed based on the definitions provided with the 5C model and the general themes emerging in the evidence, as shown in Table S2. Themes were also categorised as demonstrating vaccine hesitancy or confidence. As one of the subobjectives of this review was to capture attitudes towards vaccination following COVID-19, COVID-19 routine (e.g., recommended vaccines such as the measles vaccine), and elective (e.g., optional vaccines such as the influenza vaccines in certain age groups) vaccine data were categorised and presented separately.

All studies were subject to a quality and risk of bias assessment, using the Joanna Briggs Institute (JBI) Critical Appraisal tools. Quality/bias assessments for all publications were performed by one researcher, with discrepancies being resolved by a senior researcher.

## 3. Results

### 3.1. Summary of Results

**Figure 2:**
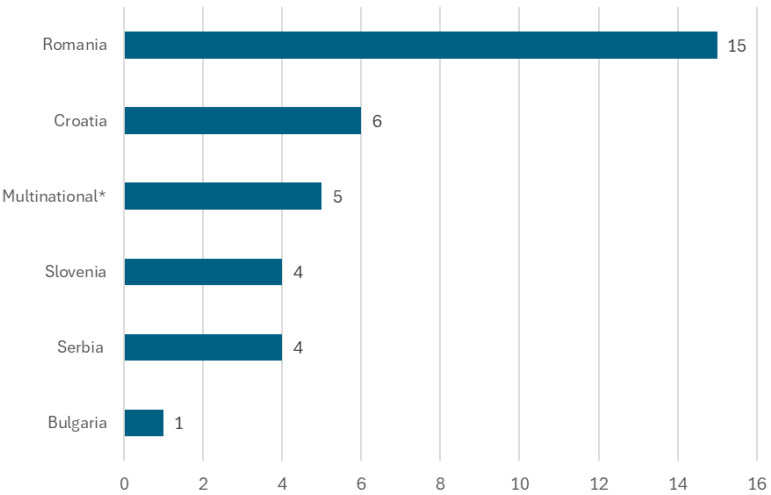
Geographic distribution of the 35 publications identified through database searches. * Included multiple countries, including Romania, Bulgaria, Croatia, Slovenia, and Serbia.

A total of 68 publications were included through the screening process; 35 were identified through databases, while 33 were identified from the grey literature. Of the 35 publications identified through databases, the majority were conducted in Romania (*n* = 15) and Croatia (*n* = 6). As some studies were conducted in multiple countries, the sum of studies identified from each Southeastern European country does not equal the total number of included studies, which is presented in Figure 2.

**Figure 3:**
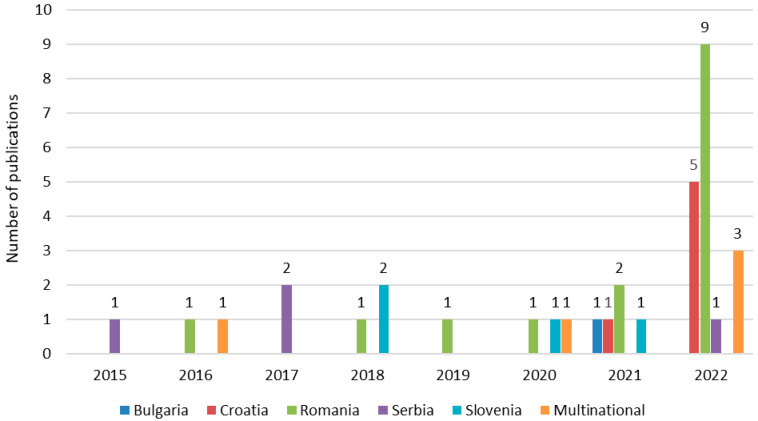
Distribution of the 35 publications identified through database searches stratified by country and year.

The most common study design utilised was cross-sectional (*n* = 31), and the remaining studies were qualitative (*n* = 4). In addition, 18 of the 35 publications (51.4%) identified through database searches were published in 2022 (Figure 3). Based on JBI Critical Appraisal Tools, the quality of these studies ranged from high to moderate, with most of the studies being of high quality. The main cause of risk of bias in cross-sectional studies included in this review was the absence of confounding factors and strategies to deal with them. For included qualitative studies, the main cause of risk of bias was the absence of statements addressing the influence of the researcher on the study.

### 3.2. Factors of Vaccine Hesitancy and Confidence in Romania

**Figure 4:**
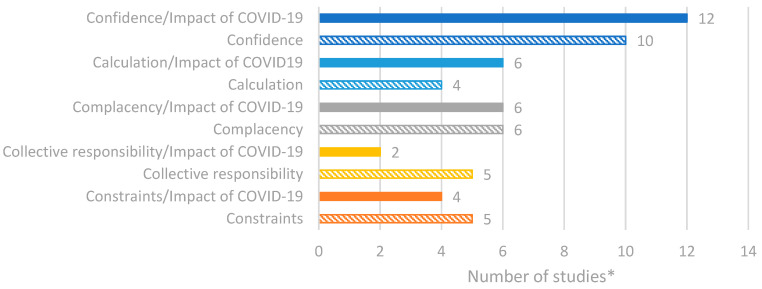
Number of studies reporting factors of vaccine hesitancy/confidence in Romania based on the 5C model. * Number of studies presented in the chart does not equate to total number of studies identified, as some studies reported multiple outcomes.

Seventeen studies were identified through the database searches that focused on vaccine hesitancy and confidence in Romania. The extracted study data were categorised according to Table S2. A summary of the reported data is presented in Figure 4. Overall, the most reported factors of vaccine hesitancy included confidence and complacency. Among studies reporting the impact of COVID-19, a similar trend was observed, with calculation—which is the individual’s engagement in extensive information searching regarding vaccination—also being identified as a factor for vaccine hesitancy [24,25,26].

### 3.3. COVID-19 Vaccines

The most reported cause of vaccine hesitancy among the Romanian population was confidence, particularly the lack of trust in COVID-19 vaccines in terms of their effectiveness and safety. Lack of trust in vaccine safety was reported in a cross-sectional study conducted from February to March 2021 [24]. The study surveyed 1552 Romanian residents aged 25–44 years, of which 29.5% expressed opposition to the COVID-19 vaccination [24]. Among the 458 respondents unwilling to receive COVID-19 vaccination, the main justification for refusal was that the COVID-19 vaccine was insufficiently safe and that there was a possibility of serious side effects (84.4%) [24]. Another cross-sectional study, conducted in November 2021, surveyed 650 unvaccinated Romanians and also reported that the lack of trust in COVID-19 vaccines was the main reason why people did not get vaccinated. The study also identified that the participants perceived COVID-19 vaccines as experimental vaccines [27]. Furthermore, a survey conducted among 2297 adults aged 18 to 66 years and over between January and March 2022 during the fifth wave of COVID-19 found that while the majority of the participants placed high confidence in mandatory childhood vaccinations (98.6%) and elective vaccines such as hepatitis A and B, influenza, meningococcal, and human papillomavirus [HPV] (97.8%), acceptability was lower with COVID-19 vaccines, with only 68.3% of the participants considering them as mandatory vaccinations, highlighting potential vaccine hesitancy. Acceptability was also lower in younger age groups, with only 58.2% of participants aged 18 to 20 years considering COVID-19 vaccines as mandatory [28]. Similar observations were identified from a cross-sectional survey conducted in October and November 2021 during the peak of the fourth COVID-19 wave among 581 Romanians who had children aged 12–18 years and who did not vaccinate their children against COVID-19. The main reasons behind refusal were that 47.3% ‘considered COVID-19 vaccines too new and that they needed to be studied more’, while 24.4% were ‘afraid of the adverse reactions of the COVID-19 vaccines’ [29].

Other causes of COVID-19 vaccine hesitancy reported in the literature included a lack of trust in healthcare. One survey conducted during the fourth wave of the COVID-19 pandemic identified that 74% of the participants did not trust the medical system in Romania, and 34% did not trust HCPs [27]. The study also found that 54.7% of the participants agreed with fake news that claims ‘vaccination on a global scale aims to enrich vaccine manufacturers’. In total, 47% of the participants also agreed that ‘there is a global secret society who wants to control the world’, while only 35.5% disagreed with this statement [27]. Interestingly, one survey conducted in May 2021 reported that among 1552 Romanian residents, 71.8% of the participants who did want to get vaccinated mainly identified the government and medical staff as their main source of information regarding COVID-19 infection (for both, *p* < 0.05) [24]. Those who did not want to get vaccinated chose to get their information from traditional media (television, newspapers and radio broadcasting) and the internet (e.g., blogs, news websites, and social media) [24,26]. Similar data was also identified among pregnant women [25].

#### Routine and Elective Vaccinations

Similar to the data on COVID-19, confidence, particularly trust in vaccines and trust in their effectiveness and safety, was the most reported outcome for routine and elective vaccinations. However, in contrast to COVID-19 data, studies reporting on routine vaccinations highlighted more positive attitudes towards vaccination. As previously highlighted, a 2022 survey conducted among 2297 adults aged 18 to 66 years and over found that 100–94.7% of the participants trusted mandatory childhood vaccinations, and 100.0–91.8% of the participants trusted elective vaccines such as hepatitis A and B, influenza, meningococcal, HPV, with trust being highest among participants aged 61–65 years [28]. However, while trust was seemingly high, a survey conducted from October to December 2014 on 918 parents identified that 64.4% refused to vaccinate their daughters against HPV, highlighting potential reluctance [30]. One ethnographic study identified through the grey literature explored the barriers and drivers of measles vaccine uptake from the perspective of caregivers and HCPs. This study was conducted from February to April 2019 and reported that ‘Fear of adverse events was identified as a barrier to vaccination’ in caregivers [31]. Furthermore, one survey conducted from October to December 2014 highlighted that Romanian parents rely on HCPs as well as internet browsing as sources of information on human papillomavirus (HPV) infection and vaccination [30].

### 3.4. Factors of Vaccine Hesitancy and Confidence in Croatia

**Figure 5:**
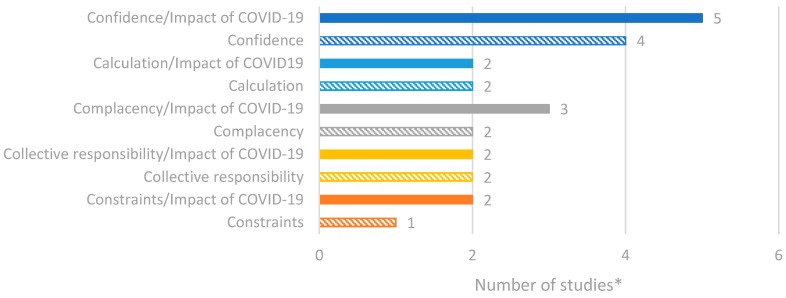
Number of studies reporting factors of vaccine hesitancy/confidence in Croatia based on the 5C model. * Number of studies presented in the chart does not equate to total number of studies identified, as some studies reported multiple outcomes.

Nine studies were identified through the database search that focused on vaccine hesitancy and confidence in Croatia. A summary of all reported data is presented in Figure 5. Overall, the most reported factors of vaccine hesitancy included confidence and complacency. Among studies reporting the impact of COVID-19, a similar trend was observed [32,33,34].

### 3.5. COVID-19 Vaccines

The most reported cause of vaccine hesitancy among the Croatian population was confidence and a lack of trust in the effectiveness and safety of COVID-19 vaccines. A key finding identified through a cross-sectional survey conducted from March to April 2021 was that 35.3% of the 765 respondents (the sample was nationally representative and respondents were aged between 18 and 88 years) stated that they were not willing to be vaccinated against COVID-19 [32]. Additionally, the predominant reasons for vaccine hesitancy were the beliefs that COVID-19 vaccines were not sufficiently safe (82.0%), only naturally acquired immunity offered true protection (72.0%), and not believing the vaccine was sufficiently effective at protecting against COVID-19 (66.6%) [32]. The grey literature also supported this, with an Al Jazeera news article published in 2020 reporting that COVID-19 vaccine hesitancy in Croatia was due to the public not believing ‘in the new vaccine’ or being ‘afraid of side effects’ [35]. Furthermore, negative attitudes towards vaccination were exacerbated in pregnant women [33]. This was demonstrated in a survey that included 430 pregnant Croatian women from May to October 2021, where 85% of women agreed that COVID-19 vaccines were not safe for unborn children [33]. Most of the women with this belief planned not to receive the COVID-19 vaccination compared to those who did (*p* < 0.00001), further emphasising lack of trust in safety as a major deterrent to COVID-19 vaccination in Croatia [33].

#### Routine and Elective Vaccinations

A major theme identified in studies exploring routine and elective vaccinations was that trust in vaccine effectiveness and safety was generally high in Croatia, particularly among HCPs [34]. This was shown in a cross-sectional survey conducted between July and December 2021 on 143 physicians and 181 nurses [34]. When asked to assess the item ‘Vaccines are useful’ on a 5-point Likert scale (1 = strongly disagree; 5 = strongly agree), physicians and nurses scored means of 4.79 and 4.26 (*p* < 0.001), respectively [34]. For the item ‘Vaccines are safe’, physicians and nurses scored means of 4.21 and 3.99 (*p* < 0.001), respectively. The study also found that 17% of HCPs were vaccine-hesitant, with a significant difference observed between physicians and nurses (7% vs. 24.9%) (*p* < 0.001) [34].

Themes of vaccine complacency among HCPs were also identified among studies reporting on routine and elective vaccinations. One survey-based study conducted from July to December 2018 identified that among 129 nurses and 77 physicians who refused flu vaccination, the main reason was due to the belief that they were protected by constant exposure (38.0% of nurses and 41.0% of physicians) [34]. Another major reason behind vaccine refusal in nurses was the belief that they had no medical indication (20.0%), and in physicians, 20.1% forgot to get vaccinated [34]. Thus, these findings suggest flu vaccine refusal among Croatian HCPs may stem from perceived risks of the flu being low [34]. However, it should be noted that these findings were derived from HCPs who refused vaccination. In general, this study showed that Croatian HCPs do not underestimate the risks of flu [34]. When all 181 nurses and 143 physicians were asked to assess the item ‘It is better to develop immunity by naturally overcoming the disease than by vaccination’ on a 5-point Likert scale (1 = strongly disagree; 5 = strongly agree), the physicians and nurses scored means of 1.96 and 2.56 (the difference between the physicians and nurses was statistically significant, *p* < 0.001), respectively [34]. Additionally, this study implied that HCPs have a heightened perception of risk towards vaccine-preventable infections in children [34]. For example, for the item ‘Vaccination of children should remain mandatory’, physicians and nurses scored means of 4.62 and 4.43 (*p* < 0.001), respectively [34].

### 3.6. Factors of Vaccine Hesitancy and Confidence in Bulgaria

**Figure 6:**
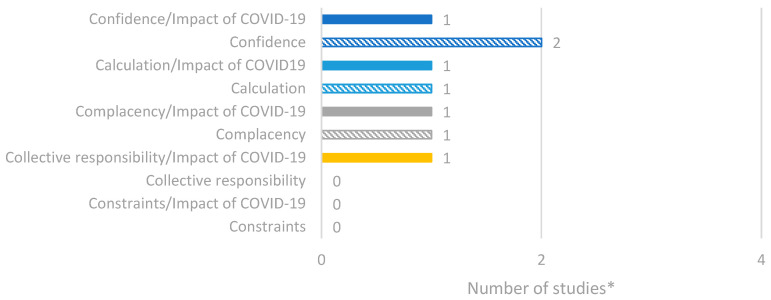
Number of studies reporting factors of vaccine hesitancy/confidence in Bulgaria based on the 5C model. * Number of studies presented in the chart does not equate to total number of studies identified, as some studies reported multiple outcomes.

Three studies were identified through the database search that focused on vaccine hesitancy and confidence in Bulgaria. A summary of the reported data is presented in Figure 6. Overall, the most reported factor of vaccine hesitancy was confidence, particularly lack of trust in the effectiveness and safety of vaccines [36,37,38].

### 3.7. COVID-19 Vaccines

A lack of trust in the effectiveness and safety of COVID-19 vaccines was identified as a key factor in vaccine hesitancy among the Bulgarian population. This was demonstrated in a multinational study, which conducted an online survey in June 2021 across Germany, the United Kingdom (UK), Sweden, Spain, France, Italy, Poland and Bulgaria. The study found that the main reason underlying vaccine hesitancy among 784 unvaccinated Bulgarian residents was fear of side effects (32.0%) [37]. Compared to Spain, where 6.22% of women and 6.82% of men were hesitant to receive the COVID-19 vaccine, Bulgaria showed the highest rates of COVID-19 vaccine hesitancy, with 64.19% of women and 59.20% of men hesitant to receive the vaccination [37]. These findings were supported by grey literature publications. For example, in a 2022 news article published by Euronews, one Bulgarian individual expressing vaccine hesitancy stated that ‘I don’t understand the statistics in the light of vaccination. I don’t see the efficacy’ [38]. One study also demonstrated that 32.0% of Bulgarian residents believed side effects associated with COVID-19 vaccination were downplayed by health authorities, therefore highlighting a potential lack of trust in healthcare [37].

#### Routine and Elective Vaccinations

Similar to COVID-19 vaccines, a lack of trust was identified in the effectiveness and safety of routine and elective vaccines [36]. For example, a cross-sectional study utilising semi-structured questionnaires was conducted from November 2016 to February 2017 and found that among 485 unvaccinated members of the Bulgarian public, the most frequent reason for not vaccinating against influenza was the belief that the vaccine was not effective: 27.6% [36]. However, more than half of the respondents (51.1%) were willing to change their attitude towards influenza vaccination, and the recommendation and advice from a doctor were the most important for them (40.8%) [36].

### 3.8. Factors of Vaccine Hesitancy and Confidence in Serbia

**Figure 7:**
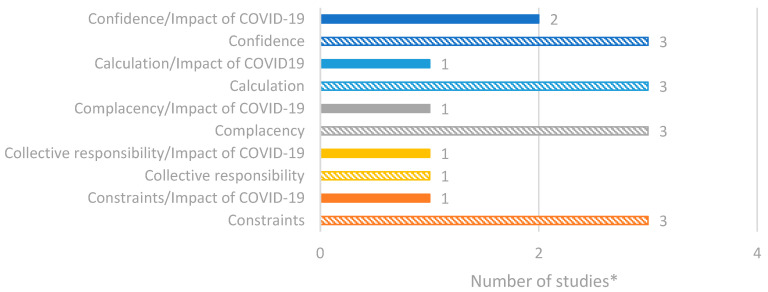
Number of studies reporting factors of vaccine hesitancy/confidence in Serbia based on the 5C model. * Number of studies presented in the chart does not equate to total number of studies identified, as some studies reported multiple outcomes.

Five studies were identified through the database search that focused on vaccine hesitancy and confidence in Serbia. A summary of all reported data is presented in Figure 7. Overall, the most reported factors of vaccine hesitancy were confidence, complacency, constraints and calculation [39,40,41,42].

### 3.9. COVID-19 Vaccines

A number of studies were identified that explored trust in COVID-19 vaccines. Interestingly, a multinational study conducted from April to May 2020 found that among 94 Serbian HCPs, only 13.8% and 11.7% disagreed with the statements ‘I believe the vaccine will be effective’ and ‘I believe the vaccine will be safe’, respectively, whereas 14.8% and 20.4% of Croatian HCPs disagreed with these statements [42]. In support of this, the same study reported that COVID-19 vaccine refusal was relatively low among Serbian HCPs (8.5%) compared to countries such as Croatia, where 12.2% of HCPs were against the use of vaccines (*p* = 0.001) [42]. However, the majority of Serbian HCPs (55.3%) were unsure about the effectiveness of the vaccine [42]. Comparatively, the grey literature reported higher COVID-19 vaccine refusal among 1200 members of the Serbian public in 2020, with 31.0% who did not want to be vaccinated against COVID-19 [43].

#### Routine and Elective Vaccinations

A major factor identified in studies exploring routine and elective vaccinations was complacency, and the data highlighted HCPs’ perceived views on parents’ attitudes towards childhood vaccinations. One cross-sectional study conducted between March and April 2012 that included Serbian paediatricians highlighted that 49.2% (*n* = 63) of participants believed parents had a negative attitude towards vaccinating their child against HPV [39]. Additionally, from the paediatricians’ perspective, some of the reasons behind parents refusing to vaccinate their daughters against HPV were the following: ‘She is still young’, ‘She is not even in high school’, and ‘there is no risk for my child to be infected’, therefore suggesting that the parents’ perceived risk of HPV may be low, and thus, they do not deem vaccination as a necessary preventative action [39]. Similar findings were reported in a cross-sectional study that included Serbian gynaecologists [40]. Furthermore, a 2017 publication of a cross-sectional study including 352 HCPs identified that ‘threat of disease’ caused the highest proportion of variance among those who refused vaccination [41]. The highest-ranked reasons for hepatitis B vaccine refusal included ‘not at increased risk’ and ‘concern about side effects of vaccine’, which highlights that the perceived risk of hepatitis B and confidence in vaccinations may be low [42].

Lack of education and financial constraints were also identified as factors of vaccine hesitancy, as reported in two cross-sectional studies from 2012 and 2014, with 46.5–38.5% of Serbian HCPs agreeing that they considered themselves insufficiently informed about HPV vaccines [39,40]. Similarly, only 39.3% (*n* = 46) of gynaecologists agreed that parents thought they were adequately informed about the HPV vaccine [40]. Furthermore, gynaecologists were reported to believe that a major reason behind parents refusing to vaccinate their children against HPV was due to HPV vaccines being ‘really expensive’ (*n* = 51, 43.6%) [40]. Likewise, the most common barrier to paediatricians recommending the HPV vaccine was reportedly its high cost, with 62.7% of 52 Serbian paediatricians who did not recommend the HPV vaccine to their patients agreeing that they considered the vaccine ‘very expensive’ [39].

### 3.10. Factors of Vaccine Hesitancy and Confidence in Slovenia

**Figure 8:**
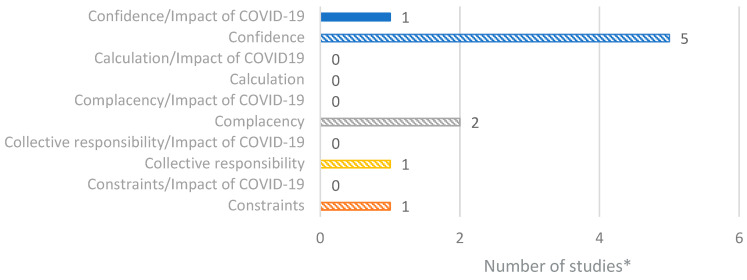
Number of studies reporting factors of vaccine hesitancy/confidence in Slovenia based on the 5C model. * Number of studies presented in the chart does not equate to total number of studies identified, as some studies reported multiple outcomes.

Six studies were identified through the database search that focused on vaccine hesitancy and confidence in Slovenia. A summary of all reported data is presented in Figure 8. Overall, confidence and complacency were identified as key factors in vaccine hesitancy [42,44,45].

### 3.11. COVID-19 Vaccines

One study, conducted during the first wave of the COVID-19 pandemic (April–May 2020), explored vaccination intention and acceptance in relation to knowledge about COVID-19 among HCPs [42]. This multinational study identified that among 87 Slovenian HCPs, 86.2% were not against the use of COVID-19 vaccines; however, only 31% and 32.2% trusted the safety and effectiveness of the vaccine, respectively, with the majority remaining unsure about the benefits of COVID-19 vaccines, implying low confidence in the vaccines [42].

#### Routine and Elective Vaccinations

Studies exploring vaccine hesitancy and confidence in routine and elective vaccinations found hesitancy to be fairly low in the Slovenian population. One 2020 publication that included 99 Slovenian parents found that 55% were not hesitant about childhood vaccination. This rate was higher than that of Bulgaria, where only 23% of parents were not hesitant [44]. Likewise, the grey literature reported that only 16.0% of the Slovenian population did not believe in vaccination.

One cross-sectional study conducted from April to June 2016 on 1704 Slovenian mothers found that among 314 mothers who were not confident about paediatric vaccines, the most trusted information sources regarding paediatric vaccinations were friends and acquaintances (50.3%) [45]. Conversely, mothers who were completely confident in vaccines trusted physicians the most (*n* = 159, 98.8%) [45].

## 4. Discussion

Across the general public populations of Bulgaria, Croatia, Romania, and Slovenia, a lack of confidence in vaccine safety and efficacy has been identified as a primary factor contributing to both routine and elective vaccine hesitancy [24,27,32,33,36,37,42]. Conversely, in Serbia, the most reported vaccine hesitancy-related factors include complacency and financial constraints [39,40]. Ultimately, the heterogeneous reasoning behind vaccine hesitancy across the different Southeastern European countries explored in this review suggests different psychological, socioeconomic and cultural reasons behind individuals’ hesitancy towards vaccination.

Although studies comparing vaccine hesitancy between paediatric and adult vaccinations were not necessarily directly comparable, our findings did demonstrate that overall, negative public perception of COVID-19 vaccination was typically increased with regard to paediatric versus adult vaccination, particularly in Romania and Croatia [24,32,33]. Safety and tolerability concerns regarding the effect of COVID-19 vaccination on paediatric and adult populations alike were prevalent, with the safety of unborn children being of particular concern for pregnant women [24,32,33]. Similarly, our findings also highlight the influence that the opinions of parents may have on general paediatric immunisation in Southeastern Europe. Studies identified across Bulgaria, Romania, Serbia, and Slovenia reported hesitancy rates of 43–77% among parents with regard to childhood vaccinations, including HPV [30,39,40,44]. Reasons for vaccine refusal included financial burden associated with having their child vaccinated, and a perceived low risk of infection for their children [30,39,40,44]. Paediatric patients not vaccinated against HPV remain vulnerable to HPV-related cancers and precancers, which may necessitate intensive treatments including chemotherapy, radiotherapy, and surgical interventions [46]. Therefore, education initiatives targeting parents in Southeastern Europe must clearly communicate the risks associated with non-vaccination and emphasise the protective benefits of childhood vaccination for preventable diseases.

A small proportion of Romanian HCPs, particularly nurses, reportedly refused vaccination for influenza due to the belief that they were protected by constant exposure. Nevertheless, vaccine acceptance was generally higher across HCPs than the general public, as observed in Croatia, Slovenia and Serbia. In addition, HCPs were observed to have a heightened perception of risk towards vaccine-preventable infections in children, with many agreeing that vaccination of children should remain mandatory [34]. Although our study was not powered to identify the reasons why vaccine acceptance was typically higher among HCPs than the general public, it could be hypothesised that access to higher education and increased medical communication literacy could contribute to a greater trust in vaccine mechanisms and their health benefits [47,48]. Therefore, governments and healthcare providers should implement efficient vaccination programmes and translate complex vaccination data into plain language to enhance public understanding of the importance of immunisation [48]. Furthermore, despite one study highlighting that Romanian parents rely on HCPs for information regarding HPV vaccination, another reported that a high proportion of the Romanian public distrusts the medical system (74%) and HCPs (34%) [27,30,49]. This emphasises the importance of building trust and improving communications between healthcare providers and the general public to improve vaccine confidence and potentially increase uptake of vaccinations in adults and children alike [27,48].

Evidence suggested that individuals may be more likely to use traditional media, friends, family and co-workers as their main source of information for COVID-19 vaccines if they were against COVID-19 vaccines [24,25,26]. Whereas, individuals who were supportive of vaccination were significantly more likely to obtain their information sources from the government and medical staff [24,25]. The differences noted between countries in key concerns regarding vaccine hesitancy underscore the need for tailored public health strategies that address safety and efficacy concerns in Southeastern Europe. Furthermore, different media platforms may have a substantial impact on the general public’s perception of vaccinations and thus their uptake. Future research and healthcare development plans should look to continuously gain the confidence of the general public to improve vaccine acceptance and uptake.

Changes in beliefs in vaccination following the COVID-19 pandemic appeared to be heterogeneous across the studies. Vaccine hesitancy in Bulgaria remained high, with population-level estimates of COVID-19 vaccine hesitancy reportedly the highest among Bulgarian women and men (64.2% and 59.2%, respectively) compared to other European countries. Conversely, COVID-19 may have exacerbated negative attitudes towards vaccination in Croatia, as evidenced by Croatian respondents least frequently expressing COVID-19 vaccine intention, and a lower mean vaccine acceptance score [42]. The sustained high levels of vaccine hesitancy in Southeastern Europe following the COVID-19 pandemic may pose significant risks to population health and herd immunity [50]. Therefore, these findings underscore the need for public health interventions tailored to improving vaccine confidence and, in turn, supporting immunisation coverage. In addition, the majority of relevant studies we included from database searches were published in 2022, suggesting an increased interest in vaccine hesitancy following the start of the COVID-19 pandemic.

The findings from this review are in line with previous research that identified fear of side effects and lack of trust in the quality and effectiveness of COVID-19 vaccines as major driving factors behind vaccine hesitancy [51,52,53,54]. Taking this into consideration, public health campaigns that place emphasis on communicating the thorough and extensive testing that all recommended vaccines undergo, including the COVID-19 vaccine, could decrease the public’s concerns regarding the safety and effectiveness of COVID-19 vaccines. In support of this, an experimental study published in 2021 found that transparent communication about the potential side effects of COVID-19 vaccines decreased participants’ distrust in vaccines in the short term and lessened trust in conspiracy beliefs and low trust in healthcare authorities in the long term. Moreover, another study reported that vaccine acceptance in the US could be significantly increased by informing individuals about the safety of COVID-19 vaccines. In accordance with these studies, future research could investigate the success of similar messages in Southeastern Europe. Additionally, this review identified complacency as another major determinant of vaccine hesitancy, with this sentiment also being reported in other studies. For example, previous research has reported that individuals were more likely to accept a COVID-19 vaccination if they had a high perceived risk of COVID-19. These findings suggest that individuals who intend to get vaccinated believe they may be at higher risk of experiencing the negative consequences of COVID-19 compared to individuals who do not intend to get vaccinated. Therefore, public health campaigns that highlight the risks and prevalence of viral infections may encourage vaccine acceptance.

### Strengths and Limitations

The main strength of this review is that it is a comprehensive, structured literature review that provides insight into participants’ opinions on vaccination via qualitative and quantitative data. However, there are limitations to structured literature reviews; for example, screening and data extraction were conducted by a single researcher, which may result in evidence bias.

The inclusion criteria specified data based on patient perspectives; thus, most of the included publications followed a cross-sectional study design, which may have limited the data collected. However, this approach allowed many publications reporting directly on participant attitudes towards vaccination to be captured. Additionally, there was sparsity in the data identified from Bulgaria and Slovenia compared to the other countries, which highlights gaps that future research may address.

Furthermore, despite a clear explanation on how data should be categorised (Table S2), the grouping of data was inadvertently subject to bias, especially when considering the overlap between vaccine hesitancy-related categories. Also, a 7C model is now available that builds upon the 5C model and focuses on themes of conspiracy (conspiracy thinking and belief in fake news related to vaccination) and compliance (support for societal monitoring and sanctioning of people who are not vaccinated). While the new model was not utilised in this review, our assessment of the data did focus on themes such as the impact of media and awareness of social responsibility, which allowed views on conspiracy and compliance to be explored [55].

Finally, literature reviews, per the methodology, are conducted at a specific timepoint, with this review reflecting evidence available between 1 January 2012 and 31 December 2022. Therefore, future updates to identify the relevant literature published after our study timeframe would be valuable to capture more recent evidence.

## 5. Conclusions

In conclusion, the evidence captured by this review suggests that the main cause behind vaccine hesitancy in several countries in Southeastern Europe is distrust in vaccine effectiveness and safety. Another factor that plays a lesser but still significant role in vaccine hesitancy in some Southeastern European countries, including Serbia and Croatia, is the perception that the risk of infection is low. Our findings also demonstrate that parental vaccine hesitancy significantly impacts paediatric immunisation in Southeastern Europe, with hesitant parents often refusing childhood vaccinations. Thus, the results of this study suggest public health campaigns that address low trust in vaccine safety and effectiveness could be carried out across Southeastern Europe, as lack of confidence in vaccine safety and effectiveness is a threat to vaccine acceptance in Romania, Bulgaria, Croatia, Serbia, and Slovenia. Further, the findings of this review can be used to tailor vaccination campaigns to specific factors influencing hesitancy, thereby more accurately addressing the public’s concerns in each country and potentially leading to more successful campaigns that decrease vaccine hesitancy and, in turn, increase vaccine uptake.

## Data Availability

No new data were created or analysed in this study.

## Supplementary Materials

The following supporting information can be downloaded at https://www.mdpi.com/article/10.3390/vaccines14040299/s1, Table S1: PICOTS criteria for study inclusion; Table S2: Data categorisation; Table S3: Search Terms.

## References

[1] Melovic B., Stojanovic A.J., Vulic T.B., Baynazoğlu M.E. (2021). Perceptions and Attitudes of Parents Toward Vaccination of Children in Western Balkan Countries: Trust in the Function of Improving Public Health. Balkan Med. J..

[2] MacDonald N.E., Butler R., Dubé E. (2018). Addressing barriers to vaccine acceptance: An overview. Hum. Vaccines Immunother..

[3] Liu L., Johnson H.L., Cousens S., Perin J., Scott S., Lawn J.E., Rudan I., Campbell H., Cibulskis R., Li M. (2012). Global, regional, and national causes of child mortality: An updated systematic analysis for 2010 with time trends since 2000. Lancet.

[4] World Health Organization World Immunization Week: Power of Vaccines: Still Not Fully Utilized, Says UN Health Agency. https://news.un.org/en/story/2017/04/555892.

[5] Reno C., Maietti E., Fantini M.P., Savoia E., Manzoli L., Montalti M., Gori D. (2021). Enhancing COVID-19 Vaccines Acceptance: Results from a Survey on Vaccine Hesitancy in Northern Italy. Vaccines.

[6] Fisher K.A., Bloomstone S.J., Walder J., Crawford S., Fouayzi H., Mazor K.M. (2020). Attitudes Toward a Potential SARS-CoV-2 Vaccine: A Survey of U.S. Adults. Ann. Intern. Med..

[7] Pfattheicher S., Petersen M.B., Böhm R. (2022). Information about herd immunity through vaccination and empathy promote COVID-19 vaccination intentions. Health Psychol..

[8] Graham F. (2019). Daily briefing: WHO calls out ‘vaccine hesitancy’ as top 10 health threat. Nature Briefing.

[9] Paules C.I., Marston H.D., Fauci A.S. (2019). Measles in 2019—Going Backward. N. Engl. J. Med..

[10] Wilder-Smith A.B., Qureshi K. (2020). Resurgence of Measles in Europe: A Systematic Review on Parental Attitudes and Beliefs of Measles Vaccine. J. Epidemiol. Glob. Health.

[11] UNICEF (2024). The State of the World’s Children 2023: For Every Child, Vaccination. Regional Brief: Europe and Central Asia. https://data.unicef.org/resources/sowc-2023/.

[12] European Centre for Disease Prevention and Control (2024). Measles on the Rise in the EU/EEA: Considerations for Public Health Response.

[13] European Centre for Disease Prevention and Control (2024). COVID-19 Vaccine Tracker. https://vaccinetracker.ecdc.europa.eu/public/extensions/COVID-19/vaccine-tracker.html#uptake-tab.

[14] Jeremic Stojkovic V., Cvjetkovic S., Jankovic J., Mandic-Rajcevic S., Matovic Miljanovic S., Stevanovic A., Jovic Vranes A., Stamenkovic Z. (2023). Attitudes towards COVID-19 vaccination and intention to get vaccinated in Western Balkans: Cross-sectional survey. Eur. J. Public Health.

[15] Boytchev H. (2021). COVID-19: Why the Balkans’ vaccine rollout lags behind most of Europe. BMJ.

[16] Wellcome Wellcome Global Monitor 2018—Chapter 5: Attitudes to Vaccines. https://wellcome.org/reports/wellcome-global-monitor/2018/chapter-5-attitudes-vaccines.

[17] Kurpas D., Stefanicka-Wojtas D., Soll-Morka A., Lomper K., Uchmanowicz B., Blahova B., Bredelytė A., Dumitra G.G., Hudáčková V., Javorska K. (2025). Vaccine Hesitancy and Immunization Patterns in Central and Eastern Europe: Sociocultural, Economic, Political, and Digital Influences Across Seven Countries. Risk Manag. Healthc. Policy.

[18] Stepovic M., Dragojevic Simic V., Zivanovic Macuzic I., Simic R., Vekic S., Sekulic M., Radovanovic S., Maricic M., Sorak M., Suljagic V. (2024). The last 3 decade of vaccination coverage in the Balkan and Eastern Europe countries with reference to the impact of the COVID-19 pandemic. Front. Pharmacol..

[19] European Digital Media Observatory COVID-19 Vaccination in Bulgaria and Romania: Too Easy to Blame It on “Fake News”. https://edmo.eu/publications/covid-19-vaccination-in-bulgaria-and-romania-too-easy-to-blame-it-on-fake-news/.

[20] Page M.J., McKenzie J.E., Bossuyt P.M., Boutron I., Hoffmann T.C., Mulrow C.D., Shamseer L., Tetzlaff J.M., Akl E.A., Brennan S.E. (2021). The PRISMA 2020 statement: An updated guideline for reporting systematic reviews. BMJ.

[21] Rocco T.S., Plakhotnik M.S., McGill C.M., Huyler D., Collins J.C. (2023). Conducting and Writing a Structured Literature Review in Human Resource Development. Hum. Resour. Dev. Rev..

[22] Betsch C., Schmid P., Heinemeier D., Korn L., Holtmann C., Böhm R. (2018). Beyond confidence: Development of a measure assessing the 5C psychological antecedents of vaccination. PLoS ONE.

[23] Salmon D.A., Dudley M.Z., Glanz J.M., Omer S.B. (2015). Vaccine Hesitancy: Causes, Consequences, and a Call to Action. Am. J. Prev. Med..

[24] Ionescu T.C., Fetecau B.I., Giurgiuca A., Tudose C. (2022). Acceptance and Factors Influencing Acceptance of COVID-19 Vaccine in a Romanian Population. J. Pers. Med..

[25] Citu C., Chiriac V.D., Citu I.M., Gorun O.M., Burlea B., Bratosin F., Popescu D.E., Ratiu A., Buca O., Gorun F. (2022). Appraisal of COVID-19 Vaccination Acceptance in the Romanian Pregnant Population. Vaccines.

[26] Cristea D., Ilie D.G., Constantinescu C., Fîrțală V. (2022). Acceptance, Hesitancy, and Refusal in Anti-COVID-19 Vaccination: A Cluster Analysis Aiming at the Typology behind These Three Concepts. Vaccines.

[27] Mărcău F.C., Purec S., Niculescu G. (2022). Study on the Refusal of Vaccination against COVID-19 in Romania. Vaccines.

[28] Mărcău F.C., Gheorghițoiu R., Bărbăcioru I.C. (2022). Survey upon the Reasons of COVID-19 Vaccination Acceptance in Romania. Vaccines.

[29] Manolescu L.S.C., Zaharia C.N., Dumitrescu A.I., Prasacu I., Radu M.C., Boeru A.C., Boidache L., Nita I., Necsulescu A., Medar C. (2022). COVID-19 Parental Vaccine Hesitancy in Romania: Nationwide Cross-Sectional Study. Vaccines.

[30] Voidăzan S., Tarcea M., Morariu S.H., Grigore A., Dobreanu M. (2016). Human Papillomavirus Vaccine—Knowledge and Attitudes among Parents of Children Aged 10–14 Years: A Cross-sectional Study, Tîrgu Mureş, Romania. Cent. Eur. J. Public Health.

[31] Dube E., Pistol A., Stanescu A., Butu C., Guirguis S., Motea O., Popescu A.E., Voivozeanu A., Grbic M., Trottier M. (2023). Vaccination barriers and drivers in Romania: A focused ethnographic study. Eur. J. Public Health.

[32] Bagić D., Šuljok A., Ančić B. (2022). Determinants and reasons for coronavirus disease 2019 vaccine hesitancy in Croatia. Croat. Med. J..

[33] Tatarević T., Tkalčec I., Stranić D., Tešović G., Matijević R. (2023). Knowledge and attitudes of pregnant women on maternal immunization against COVID-19 in Croatia. J. Perinat. Med..

[34] Tomljenovic M., Petrovic G., Antoljak N., Hansen L. (2021). Vaccination attitudes, beliefs and behaviours among primary health care workers in northern Croatia. Vaccine.

[35] Filipović M. The Vaccine Is at the Door, and Most People Don’t Want It. Aljazeera. https://balkans-aljazeera-net.translate.goog/teme/2020/11/23/cjepivo-pred-vratima-vecina-ga-ne-zeli?_x_tr_sl=auto&_x_tr_tl=en&_x_tr_hl=en&_%E2%80%A6.

[36] Rangelova V., Kevorkian A., Raycheva R. (2021). Knowledge, attitudes, and practices towards the influenza vaccine among adult population in Plovdiv, Bulgaria. Arch. Balk. Med. Union.

[37] Steinert J.I., Sternberg H., Prince H., Fasolo B., Galizzi M.M., Büthe T., Veltri G.A. (2022). COVID-19 vaccine hesitancy in eight European countries: Prevalence, determinants, and heterogeneity. Sci. Adv..

[38] Gomez J. (2021). Bulgaria’s Vaccine Battle: The Mistrust Driving COVID’s Surge. Euronews.

[39] Nikolic Z., Matejic B., Kesic V., Eric Marinkovic J., Jovic Vranes A. (2015). Factors influencing the recommendation of the human papillomavirus vaccine by Serbian pediatricians. J. Pediatr. Adolesc. Gynecol..

[40] Stamenkovic Z., Matejic B., Djikanovic B., Zaric M. (2017). Gynecologists’ Knowledge, Attitudes, and Intentions Toward Human Papillomavirus Vaccination in Serbia. J. Low. Genit. Tract Dis..

[41] Kisic-Tepavcevic D., Kanazir M., Gazibara T., Maric G., Makismovic N., Loncarevic G., Pekmezovic T. (2017). Predictors of hepatitis B vaccination status in healthcare workers in Belgrade, Serbia, December 2015. Eurosurveillance.

[42] Kregar Velikonja N., Globevnik Velikonja V., Verdenik I., Jurišić I., Stanisavljević S., Dobrowolska B., Erjavec K. (2022). Vaccination intention among healthcare workers during the first wave of the coronavirus disease 2019 pandemic in relation to knowledge: A cross-sectional study in Croatia, Slovenia, Serbia, and Poland. Croat. Med. J..

[43] Mihailović S. Serbian Public Opinion on COVID-19. Demostat. https://demostat.rs/sr/vesti/istrazivanja/javno-mnjenje-srbije-o-kovidu-19/1010.

[44] Hadjipanayis A., van Esso D., Del Torso S., Dornbusch H.J., Michailidou K., Minicuci N., Pancheva R., Mujkic A., Geitmann K., Syridou G. (2020). Vaccine confidence among parents: Large scale study in eighteen European countries. Vaccine.

[45] Učakar V., Fafangel M., Kraigher A. (2018). Vaccine confidence among mothers of young children, Slovenia, 2016. Vaccine.

[46] Centers for Disease Control and Prevention Impact of the HPV Vaccine. https://www.cdc.gov/hpv/vaccination-impact/index.html#:~:text=Not%20getting%20HPV%20vaccine%20leaves,)%2C%20chemotherapy%2C%20or%20radiation.

[47] Klüwer B., Gleditsch R., Rydland K.M., Mamelund S.E., Laake I. (2024). Higher educational attainment associated with higher confidence in influenza vaccination in Norway. Vaccine.

[48] Michel J.P., Goldberg J. (2021). Education, Healthy Ageing and Vaccine Literacy. J. Nutr. Health Aging.

[49] Mihai A. (2022). A Success Story in Romania’s Struggle to Control Cervical Cancer. Sharing Progress in Cancer Care. https://cancerworld.net/success-story-romanias-control-cervical-cancer/.

[50] Cascini F., Pantovic A., Al-Ajlouni Y., Failla G., Ricciardi W. (2021). Attitudes, acceptance and hesitancy among the general population worldwide to receive the COVID-19 vaccines and their contributing factors: A systematic review. eClinicalMedicine.

[51] Palm R., Bolsen T., Kingsland J.T. (2021). The Effect of Frames on COVID-19 Vaccine Resistance. Front. Political Sci..

[52] Thunstrom L., Ashworth M., Finnoff D., Newbold S. (2020). Hesitancy towards a COVID-19 vaccine and prospects for herd immunity. SSRN Electron. J..

[53] Troiano G., Nardi A. (2021). Vaccine hesitancy in the era of COVID-19. Public Health.

[54] Neumann-Böhme S., Varghese N.E., Sabat I., Barros P.P., Brouwer W., van Exel J., Schreyögg J., Stargardt T. (2020). Once we have it, will we use it? A European survey on willingness to be vaccinated against COVID-19. Eur. J. Health Econ..

[55] Rees F., Geiger M., Lilleholt L., Zettler I., Betsch C., Böhm R., Wilhelm O. (2022). Measuring parents’ readiness to vaccinate themselves and their children against COVID-19. Vaccine.

