# Supplementary material for: Vaccine Confidence and Vaccine Hesitancy in Several Countries in Southeastern Europe in Past 10 Years: A Structured Review of Published Literature
Source: Vaccines (Basel). 2026 Mar 27;14(4):299. doi: 10.3390/vaccines14040299 (PMC13120427; doi:10.3390/vaccines14040299)
Supplement: Supplementary file 1 [file vaccines-14-00299-s001.zip › vaccines-4067795-supplementary.pdf]

Table S1: PICOTS criteria for study inclusion.

PICOTS aspect	Inclusion criteria	Exclusion criteria
**Population(s)**	> Parents<br>> HCPs<br>> General public (≥17 years of age)	Children (≤16 years of age)
**Interventions**	Adult and paediatric vaccines	Vaccines under development/not publicly available (e.g., HIV vaccine)
**Comparisons**	No vaccination	N/A
**Outcomes**	To include but not restricted to:<br>> Key attitudes to vaccination*:<br>- Confidence e.g., trust in effectiveness and safety of vaccines<br>- Constraints e.g., ability to pay for vaccines, geographical accessibility, and ability to understand immunisation services<br>- Complacency e.g., perception of risks of infectious diseases<br>- Calculation e.g., engagement with available vaccine information resources<br>- Collective responsibility e.g., awareness of social responsibility<br>> Delayed vaccination<br>> Vaccination refusal<br>> Differences in attitudes to paediatric and adult vaccination<br>> Impact of the COVID-19 pandemic on attitudes towards vaccinations	Publications not reporting outcomes of interest.
**Time**	January 1st, 2012-December 31st, 2022	Publications prior to 2012
**Study design**	To include but not restricted to:<br>> Observational studies (retrospective and prospective)<br>> Epidemiological studies<br>> Government reports<br>> Official public health	> RCTs<br>> Economic models<br>> Case studies<br>> Editorials<br>> Systematic reviews<br>> Research and development articles (except those that include vaccine hesitancy)<br>> Non-peer reviewed publications

	websites<br>> PhD theses	
**Other**	Human studies only	Animal studies

* Outcomes based on the 5C model [17].

Abbreviations: HCPs, healthcare professionals, HIV, human immunodeficiency virus; N/A, Not applicable; RCTs, randomised controlled trials.

Table S2. Data categorisation.

Category*	Category definition	Subcategories
**Constraints**<br>**Constraints/Impact of COVID-19**	Structural and psychological barriers that impede the implementation of vaccination intentions into behaviour.	> Financial<br>> Accessibility<br>> Lack of education
**Collective responsibility**<br>**Collective responsibility/Impact of COVID- 19**	The willingness to protect others by one's own vaccination by means of herd immunity.	> Awareness of social responsibility
**Complacency**<br>**Complacency/Impact of COVID-19**	When perceived risks of vaccine-preventable diseases are low/high and vaccination is/is not deemed a necessary preventative action.	> Perception of risk<br>> Perception of risk/attitudes towards vaccination
**Calculation**<br>**Calculation/Impact of COVID-19**	Individuals' engagement in extensive information searching regarding vaccination.	> Engagement with available information<br>> Impact of media<br>> Impact of friends and family<br>> Impact of research<br>> Impact of HCPs<br>> Impact of government
**Confidence**<br>**Confidence/Impact of COVID-19**	Trust in the effectiveness and safety of vaccines, the system that delivers them, including the reliability and competence of the health services and health professionals, and the motivations of policymakers who decide on the need of vaccines.	> Trust in manufacturers and/ or pharmaceutical companies<br>> Trust in effectiveness and safety<br>> Trust in healthcare<br>> Trust in vaccines<br>> Trust in information sources<br>> Vaccine refusal<br>> Vaccine hesitancy<br>> Vaccine acceptance

*Data from publications were categorised into the following categories, which were broadly based on the 5C model [17].

Abbreviations: HCPs, healthcare professionals.

Table S3. Search Terms

Line	Search terms
**1**	exp Vaccination/ or exp Vaccines/
**2**	exp Vaccines, Synthetic/
**3**	exp Viral Vaccines/
**4**	exp Vaccines, Attenuated/
**5**	exp Vaccines, Subunit/
**6**	exp Vaccines, DNA/
**7**	exp Vaccines, Acellular/
**8**	exp Vaccines, Virosome/
**9**	exp Vaccines, Inactivated/
**10**	exp mRNA Vaccines/
**11**	exp Papillomavirus Vaccines/
**12**	exp Measles-Mumps-Rubella Vaccine/
**13**	exp Meningococcal Vaccines/
**14**	exp Diphtheria-Tetanus-Pertussis Vaccine/
**15**	exp Rotavirus Vaccines/
**16**	exp Pneumococcal Vaccines/
**17**	exp Influenza Vaccines/
**18**	exp Immunization/
**19**	exp Immunization/ or exp Immunization Programs/
**20**	exp Mass Vaccination/
**21**	(vaccin* or immuni* or inocul*).ti,ab.
**22**	1 or 2 or 3 or 4 or 5 or 6 or 7 or 8 or 9 or 10 or 11 or 12 or 13 or 14 or 15 or 16 or 17 or 18 or 19 or 20 or 21
**23**	exp Anti-Vaccination Movement/
**24**	exp Vaccination Hesitancy/ or exp Vaccination Coverage/ or exp Vaccination Refusal/
**25**	(barrier* or uptake or refus* or decline* or hesitan* or confidence or confident).ti,ab.
**26**	(patient$ adj2 (attitude$ or compliance or "non compliance" or adheren$ or "non adherence" or participation or "non participation" or preference$ or satisf$ or dissatisf$ or toleran$ or intoleran$ or "reported outcome" or "reported outcomes")).ti,ab.
**27**	(preference* adj3 (valu* or measur* or health or life or estimat* or elicit* or disease or score* or instrument or instruments)).ti,ab.
**28**	("vaccin* acceptance" or "delayed vaccin* acceptance" or "altered vaccin* schedule").ti,ab.
**29**	((patient or physician or parent or caregiver) adj3 (accept* or willingness)).ti,ab.
**30**	23 or 24 or 25 or 26 or 27 or 28 or 29
**31**	(exp Animal/ or nonhuman/) not exp human/
**32**	exp case study/ or exp case report/ or exp letter/ or exp preliminary communication/ or exp note/ or exp editorial/ or exp editor/ or exp editorial policies/ or exp newspaper/
**33**	22 and 30
**34**	31 or 32
**35**	33 not 34
**36**	exp Balkan Peninsula/
**37**	exp Croatia/

**38**	exp Serbia/
**39**	exp Slovenia/
**40**	exp Romania/
**41**	exp Bulgaria/
**42**	(balkan or Croatia or Serbia or Slovenia or Romania or Bulgaria).ti,ab.
**43**	36 or 37 or 38 or 39 or 40 or 41 or 42
**44**	35 and 43
**45**	limit 44 to yr="2012 -Current"
**46**	limit 45 to human
**47**	remove duplicates from 46
